# Use of past care markers in risk-adjustment: accounting for systematic differences across providers

**DOI:** 10.1007/s10198-021-01350-9

**Published:** 2021-07-31

**Authors:** Laura Anselmi, Yiu-Shing Lau, Matt Sutton, Anna Everton, Rob Shaw, Stephen Lorrimer

**Affiliations:** 1 Health Organisation, Policy and Economics, University of Manchester, Manchester, UK; 2 Analysis and Insight for Finance, NHS England and NHS Improvement, London, UK; 3 Melbourne Institute of Applied Economic and Social Research, University of Melbourne, Melbourne, Australia; 4 Analytical Services, NHS England and NHS Improvement, London, UK

**Keywords:** Risk-adjustment, Resource allocation, Weighted capitation, Need, Cost, Mental health

## Abstract

Risk-adjustment models are used to predict the cost of care for patients based on their observable characteristics, and to derive efficient and equitable budgets based on weighted capitation. Markers based on past care contacts can improve model fit, but their coefficients may be affected by provider variations in diagnostic, treatment and reporting quality. This is problematic when distinguishing need and supply influences on costs is required.

We examine the extent of this bias in the national formula for mental health care using administrative records for 43.7 million adults registered with 7746 GP practices in England in 2015. We also illustrate a method to control for provider effects.

A linear regression containing a rich set of individual, GP practice and area characteristics, and fixed effects for local health organisations, had goodness-of-fit equal to *R*^2^ = 0.007 at person level and *R*^2^ = 0.720 at GP practice level. The addition of past care markers changed substantially the coefficients on the other variables and increased the goodness-of-fit to *R*^2^ = 0.275 at person level and *R*^2^ = 0.815 at GP practice level. The further inclusion of provider effects affected the coefficients on GP practice and area variables and on local health organisation fixed effects, increasing goodness-of-fit at GP practice level to *R*^2^ = 0.848.

With adequate supply controls, it is possible to estimate coefficients on past care markers that are stable and unbiased. Nonetheless, inconsistent reporting may affect need predictions and penalise populations served by underreporting providers.

**Supplementary Information:**

The online version contains supplementary material available at 10.1007/s10198-021-01350-9.

## Introduction

Risk-adjustment models seek to predict the health care cost of individuals based on their observable characteristics with the practical purpose of attaching prospective health care premiums or budgets to them.

The aim of risk-adjustment formulae differs according to the structure of health care systems and their financing modalities. In competitive health care systems, they are used by social insurers, for example in Europe or in the US Medicare scheme, or by private insurers, to design payment systems that promote affordability and efficiency [1]. The aim is to equalise risk and compensate insurers for predictable variation in healthcare expenses across groups, incentivising the efficient provision of care and minimising adverse selection [2, 3].

In non-competitive care systems, risk-adjustment formulae are used by governments to allocate pooled resources to local strategic purchasers, according to the need of their populations, for example, in England, Canada, Australia, Norway and Sweden [4–8]. Risk and adverse selection are less of a concern because budgets are ultimately attached to a population identified by the geographic area of residence, rather than individual own risk. Policy makers and researchers are instead concerned with avoiding perverse incentives, for example related to prior service utilisation, and containing and allocating the budget equitably. Equitable allocations are intended to create a playing level field based on population need, rather than existing access to care [5, 8], while responsiveness to service users’ needs, efficiency and outcomes achievement are fostered through complementary policies targeting different levels and aspects of service delivery [4].

The most advanced risk-adjustment formulae rely on person-based models. Individual-level health care expenditure is modelled as a function of individual and area-level variables, which capture both legitimate and illegitimate sources of variation. Such distinction is crucial in systems where cross-subsidisation, or compensation through resource transfer, is used across payment plans or local health organisation budgets to address fairness concerns.

The choice of variables, along with the model predictive power, is very important for setting fair budgets [9]. Models should include a rich set of needs variables, which typically drive legitimate variation in health care expenditure, and for which cross-subsidisation is desirable [6, 10]. Variables for which cross-subsidisation is not desirable, such as those reflecting health care supply or insurer responsibilities, should be included to obtain unbiased coefficients on the need variables. Their effect can then be sterilised, for example by fixing their levels to an average value when predicting costs, so that insurers or purchasers are equally rewarded for individuals with equal need [11–14].

The distinction between need and supply variables has underpinned the methodological development of risk-adjustment formulae in non-competitive health care systems. Research on resource allocation in England is one of the most documented examples and the one with the longest tradition [4, 15].

Need variables are typically basic demographic factors (age and gender) and additional personal characteristics, which improve predictive performance, and enable disentangling need from supply [4, 10, 16]. Need indicators should be feasible, universally and consistently recorded for the population included in a register of patients, possibly universal, not vulnerable to manipulation and free from perverse incentives [1, 4].

Person-level indicators that satisfy these criteria are limited. Past care markers reported in records from previous encounters with the health system, such as recorded diagnoses and intensity of care received, substantially improve the goodness of fit [17]. For example, R-squared for diagnoses-based prospective models are 15.3% versus only 1.5% for age and sex models estimated on US commercial data [6] and 13% versus 3% in models estimated on UK population data [16].

Because the intensity of care provided and the completeness of recording may differ across providers for patients with the same intrinsic need, past care markers may reflect supply as well as need. Differences may arise from perverse responses, such as increased reported complexity in activity-based financing systems [18], or simply from variations in provider capacity. Lower capacity may lead to lower care and information reporting, especially when monitoring or incentives to record activity are weak, for example when records are not used for payments. Risk-adjustment formulae may then reward higher capacity, resulting in low capitation or premiums for those patient categories who failed to secure access to care when needed [13, 14, 19–21].

Diagnostic variables are commonly used for risk-adjustment in competitive health care systems, but past care contacts and expenditure less so [6]. All are used in person-based resource allocation formulae in England, if the quality of administrative records is considered sufficient [16, 19, 22]. With a focus on general and acute care in England, Gravelle et al. [12] showed that the inclusion of morbidity variables in area level models improved the predictive power and the precision of coefficients. However, they also showed that failure to account for unobserved variation in supply may inflate the positive association between past care markers and expenditure, and in turn need predictions as a reflection of better supply. They tested the inclusion of provider effects in their model and concluded that local purchaser dummies would suffice to control for supply variation.

Gravelle et al. [12] did not consider whether their conclusions would hold with person level data, nor discussed the potential bias arising from differences across providers in reporting and data quality, as well as treatment provision. Dixon et al. [19] found that in the formula for general and acute hospital services past care markers would reflect need rather than supply, when the latter was properly accounted for. However, concerns remain on whether supply variation is sufficiently accounted for when the quality of data is particularly poor.

In this paper, we focus on the inclusion of past care markers, diagnoses and count of care contacts in the most recent formula for mental health services in England. The variability in care provision across provider, the inequity in access to care, and the concerns about the quality of administrative data, exacerbate the potential draw-backs of using markers based on past care. The development of the resource allocation formula for mental health services and the selection of variables for risk-adjustment are described in Anselmi et al. [23]. We build on their model.

We highlight the trade-off between using past care markers in a formula set to generate fair allocations, but that in practice may reflect undesired differences across providers, if they differ in diagnosing and reporting. We derive and discuss the probability limits of the coefficients for a set of alternative models in a way similar to how Gravelle et al. [12] did to illustrate the consequences of including additional controls for socio-economic status, morbidity, waiting times and provider characteristics in area level models. We then illustrate how the inclusion of past care markers improves predictive power and reduces bias in the coefficients on remaining variables. We formally assess the stability of coefficients by including additional area level measures of past care contacts with specific providers (provider effects) [24], along with fixed effects for local health organisations. This allows us to control for differences across providers serving populations within the same local health system. We assess bias and improvement in predictive power simultaneously, applying a method proposed by Oster [25].

## Health care, resource allocation and risk-adjustment in England

### National Health Service organisational structure and resource allocation

Under the current organisational structure, the health care budget in England is allocated to local health organisations, called Clinical Commissioning Groups (CCGs). These strategic purchasers commission care from primary, community and secondary care providers. CCGs have been combined into Sustainability and Transformation Partnerships (STPs) and now in Integrated Care Systems (ICS). ICSs cover all of England and are made of NHS organisations and councils, which collaborate to improve health and care in the areas they serve. The majority of health care budget is allocated to CCGs, except for primary care and for some specialised services which are commissioned centrally. The budget for primary care is mostly allocated directly to General Practitioner (GP) practices, which provide primary care and refer patients for secondary care. Every GP practice is part of one CCG and patients register with a single GP practice.

Since 1976, weighted capitation formulae have been developed in England to ensure that the distribution of resources across local health organisations reflects relative need. A set of formulae, one for each identified funding stream, are used to estimate the determinants of health care costs and to derive need indices and need-based budget shares. These are then aggregated to determine each local health organisation’s fair share of the total budget [26–28].

### Risk-adjustment development and use in resource allocation

Since 2011, person-based formulae have been applied to individual-level data covering health care users and non-users registered with GP practices [16, 22]. Each formula estimates the individual cost for service use in a given year, as a linear function of person and area level need variables and of area, GP practice and local health organisations supply variables in the previous two years. The estimated coefficients are then used to predict individual cost. Importantly, the effects of the supply variables, including local organisation dummies and provider effects, are neutralised in this cost prediction by fixing the level of supply, usually at the average value across the country. The individual-level predictions, which reflect only variations in the needs variables, are then aggregated to area or GP practice level.

Person level data used in most recent formulae typically include demographic characteristics (age and gender) for each patient registered with a GP practice, as well as information from linked administrative data sources. Information on individual ethnicity, past service use and diagnoses reported during these past contacts, is linked in via an individual patient identifier, for those who have been in contact with services and for whom administrative records exist. Information on need and supply characteristics at the GP practice and small area level, which is available from different sources, can also be linked.

### Mental Health risk-adjustment and data

The mental health formula that we use here for illustration was one of the formulae which informed the 2019/2020 round of budget allocations across CCGs [23]. In 2016, the year of the analysis, there were 211 CCGs responsible for commissioning from 66 providers (Mental Health Trusts) secondary inpatient and community mental health care for patients registered with the GP practices in their area. The GP practices were responsible for referring patients to these secondary mental health care providers.

A separate formula for mental health services was first introduced in 1996 [29] and updated multiple times broadening the scope and developing the methods towards a person-based formula [30, 31]. The formula estimated in 2012 [31] included 43 flags for three-digit ICD10 diagnostic codes, condition severity (measured in layers of at least *n* contacts with *n* different care professionals) and care markers in the two previous years, derived from the national mental health dataset [31]. Due to concerns around data quality, past care markers were not included in the latest version of the formula that we use for illustration [23].

Mental Health Trusts report information on every care contact through the national Mental Health Services Dataset (MHSDS), previously Mental Health Minimum dataset (MHMDS)[32] and Mental Health and Learning Disabilities Dataset (MHLDDS) [33]. The MHSDS data collection started in 2003 under different names (Mental Health Minimum dataset MHMDS [32] first and then Mental Health and Learning Disabilities Dataset MHLDDS [33]). The MHSDS has been broadening in scope over time including patient characteristics, diagnoses and the quantity and type of care provided. The quality of recording is improving [34, 35], but still thought to be generally low and very heterogeneous across providers [36]. This means that not only patients with the same underlying need may receive different levels of care, but also that their diagnostic and care contacts information may be reported by providers to different extents. Therefore, patients served by a provider that does not record information will appear as having no diagnoses and no multiple care contacts. For example, providers differ in clustering patients by need, but these differences are not systematically associated with any observable provider characteristic [36].

## Methodological issues

### A model for mental health care cost

In line with previous work [12, 16, 30, 31, 37], we assume that the cost of mental health care is a function of observed and unobserved need and supply variables at the individual, GP practice and CCG level

1 $${C}_{ijk}\,=\,{\beta }_{0}+{\beta }_{1}{x}_{1 ijk}+{\beta }_{2}{x}_{2 ijk}+{\beta }_{3}{x}_{3ijk}+{\beta }_{4}{x}_{4jk}+{\beta }_{5}{z}_{jk}+{\beta }_{6}{P}_{jk}+{\beta }_{7}{K}_{k}+{\varepsilon }_{ijk},$$

where *C*_*ijk*_ is the total cost for individual *i* registered with GP practice *j*, in CCG *K.*

Need is captured by a set of individual variables, some observable (*x*_*1ijk*_ and *x*_*3ijk*_) and other not observable (*x*_*2ijk*_), and GP practice variables *x*_*4jk*_. *x*_*1ijk*_ typically include demographic and other characteristics measurable at the individual level, while *x*_*4jk*_ include proxies for need not available at the individual level, such as area deprivation. For simplicity, we refer to *x*_*4jk*_ as GP practice variables, but area level variables are usually also included in an analogous manner.

*x*_*3ijk*_ include variables, such as past care markers, which are observed only for those individuals who had previously been in contact with mental health services, and for whom the information about the contact was reported in the administrative records. The observed past mental care markers may therefore depend on individual need, as well as on the propensity of a GP practice to refer patients, on the diagnostic and treatment intensity, and on the capacity and recording precision of the provider. *x*_*3ijk*_ can therefore be written as a function of the remaining need and supply variables:

2 $${x}_{3 ijk}={\varphi }_{0}+{\varphi }_{1}{x}_{1 ijk}+{\varphi }_{2}{x}_{2 ijk}+{\varphi }_{3}{x}_{4jk}+{\varphi }_{4}{z}_{jk}+{\varphi }_{5}{P}_{jk}+{\varphi }_{6}{K}_{k}+{\varepsilon }_{ijk}^{x3}.$$

Health care supply is captured by a set of observed GP practice characteristics (*z*_*jk*_) and by referral to different mental health care providers *P*_*jk*_. *P*_*jk*_ is a set of provider effects (*p*^*m*^_*jk*_), the proportion of individuals registered with a GP practice *j* who received care from provider *m*:

3 $${p}_{jk}^{m}= \frac{\sum_{j}{u}_{ijk}^{m}}{{N}_{jk}}= \frac{\sum_{j}{u}_{ijk}}{{N}_{jk}}* \frac{\sum_{j}{u}_{ijk}^{m}}{\sum_{j}{u}_{ijk}}.$$

*N*_*jk*_ is the total number of patients registered with GP practice *j* in CCG *k*. *u*_*ijk*_ and *u*^*m*^_*ijk*_ are binary indicator taking value 1 if individual *i* has had at least one care contact with any provider or with provider *m* specifically in the two previous years, and 0 otherwise.

Each *p*^*m*^_*jk*_ is the product of the proportions of individuals registered with a GP practice who had at least one mental health care contact with any provider and the proportion of them who have had at least one contact with provider *m*. Hence, *p*^*m*^_*jk*_ reflects both the probability of receiving mental health care and receiving care from a specific provider *m* for a patients in GP practice *j*. As most GP practice lists include over a thousand patients, the contribution of *i* to *p*^*m*^_*jk*_ is minimal, and *p*^*m*^_*jk*_ can be considered exogenous to *i*.

*P*_*jk*_ is a function of GP practice need and supply variables, including a combination of unobserved characteristics of the GP practice and providers (*s*_*jk*_):

4 $${P}_{jk}={\xi }_{0}+{\xi }_{1}{x}_{4jk}+{\xi }_{2}{z}_{jk}+{\xi }_{3}{s}_{jk}+{\xi }_{4}{K}_{k}+{\varepsilon }_{jk}^{P}.$$

*s*_*jk*_ captures both the unobserved GP practice propensities to refer patients for mental health care and to each provider *m* more specifically. Without affecting the illustration of the bias mechanism, we ignore for simplicity that *P*_*jk*_ also depends on the GP practice average of individual need variables.

Whilst most GP practices would refer patients to the same mental health provider, local purchasers cover larger areas and multiple GP practices, so their patients could be referred to one or multiple providers. If all GP practice patients were in touch with the same provider, conditional on observed need, *P*^*m*^_*jk*_ would pick-up any unobserved GP practice variation in supply. *P*_*jk*_ control for unobserved differences in diagnostics intensity, care provision and recording across combinations of GP practices and providers (*s*_*jk*_). *P*_*jk*_ serve the role of provider dummies, but because not every patient uses the service and because patients can use multiple providers, *p*^*m*^_*jk*_ vary between 0 and 1.

*K*_*k*_ is a set of CCG dummy variables, which account for CCG factors, such as differences in prices or service commissioning, if any, and mean intensity of care. *K*_*k*_ account for differences in supply rather than need, and arguably, if provider effects are not included, for average differences in care provision, diagnostic intensity and data reporting across providers serving the area.

*ε*_*ijk*_ is a zero mean error uncorrelated with need, supply and CCG variables.

The constant term corresponds to a fixed average capitation rate for all registered patients, conditional on need and supply. When all variables have mean zero, the relative size of this term determines the relative size of funds that would be distributed on a purely per capita as opposed to a need basis.

### Empirical models

To illustrate how omitted variable bias may affect the coefficients of the need and supply variables when past care markers and provider effects are or are not included, we estimate the following four alternative models.

#### Model 1: without past care markers and without provider effects

First, we consider the true cost as a function of need and supply variables, excluding past care markers and provider effects, as it is common practice in more conservative person-based models.

We replace Eqs. 2 and 4 into Eq. 1 and we obtain:

5 $$ \begin{aligned} C_{ijk} & = \left( {\beta_{0} + \beta_{3} \varphi_{0} + \left( {\beta_{3} \varphi_{5} + \beta_{6} } \right)\xi_{0} } \right) + \left( {\beta_{1} + \beta_{3} \varphi_{1} } \right)x_{1 ijk} + \left( {\beta_{2} + \beta_{3} \varphi_{2} } \right)x_{2 ijk} \\ & \quad + \left( {\beta_{3} \varphi_{3} + \beta_{4} + \left( {\beta_{6} + \beta_{3} \varphi_{5} } \right)\xi_{1} } \right)x_{4jk} + \left( {\beta_{3} \varphi_{4} + \beta_{5} + \left( {\beta_{3} \varphi_{5} + \beta_{6} } \right)\xi_{2} } \right)z_{jk} + \left( {\left( {\beta_{3} \varphi_{5} + \beta_{6} } \right)\xi_{3} } \right)s_{jk} \\ & \quad + \left( {\left( {\beta_{3} \varphi_{5} + \beta_{6} } \right)\xi_{4} + \beta_{3} \varphi_{6} + \beta_{7} } \right)K_{k} + \left( {\left( {\beta_{3} \varphi_{5} + \beta_{6} } \right)\varepsilon_{jk}^{P} + \beta_{3} \varepsilon_{ijk}^{x3} + \varepsilon_{ijk}^{{}} } \right), \\ \end{aligned} $$

which we can estimate as:

6 $${C}_{ijk}={d}_{0}+{d}_{1}{x}_{1 ijk}+{d}_{2}{x}_{4jk}+{d}_{3}{z}_{jk}+{d}_{4}{K}_{k}+{\varepsilon }_{ijk}^{d}.$$

#### Model 2: with past care markers and without provider effects

Second, we consider the past care markers as observed need variables. We replace Eq. 4 into Eq. 1 and we obtain:

7 $${C}_{ijk}\,=\, \left({\beta }_{0}+{{\beta }_{6}\xi }_{0}\right)+ {\beta }_{1}{x}_{1 ijk}+{\beta }_{2}{x}_{2 ijk}+{\beta }_{3}{x}_{3 ijk}+\left({\beta }_{4}+{\beta }_{6}{\xi }_{1}\right){x}_{4jk}+\left({\beta }_{5}+{\beta }_{6}{\xi }_{2}\right){z}_{jk}+{\beta }_{6}{\xi }_{3}{s}_{jk}+\left({\beta }_{6}{\xi }_{4}+{\beta }_{7}\right){K}_{k}+\left({{\beta }_{6}\varepsilon }_{jk}^{P}+{\varepsilon }_{ijk}\right),$$

which can be estimated as:

8 $${C}_{ijk}={g}_{0}+{g}_{1}{x}_{1 ijk}+{g}_{2}{x}_{3ijk}+{g}_{3}{x}_{4jk}+{g}_{4}{z}_{jk}{+g}_{5}{K}_{k}+{\varepsilon }_{ijk}^{g}.$$

#### Model 3: without past care markers and with provider effects

We then consider the provider effects as observed supply variables. We replace Eq. 2 into Eq. 1 and we obtain:

9 $${C}_{ijk}=\left({\beta }_{0}+{\beta }_{3}{\varphi }_{0}\right)+\left({\beta }_{1}+{\beta }_{3}{\varphi }_{1}\right){x}_{1 ijk}+\left({\beta }_{2}+{\beta }_{3}{\varphi }_{2}\right){x}_{2 ijk}+\left({\beta }_{3}{\varphi }_{3}+{\beta }_{4}\right){x}_{4jk}+\left({\beta }_{3}{\varphi }_{4}+{\beta }_{5}\right){z}_{jk}+\left({\beta }_{3}{\varphi }_{5}+{\beta }_{6}\right){P}_{jk}+\left({\beta }_{3}{\varphi }_{6}+{\beta }_{7}\right){K}_{k}+{\beta }_{3}{\varepsilon }_{ijk}^{x3}+{\varepsilon }_{ijk},$$

which can be estimated as:

10 $${C}_{ijk}={a}_{0}+{a}_{1}{x}_{1 ijk}+{a}_{2}{x}_{4jk}+{a}_{3}{z}_{jk}+{a}_{4}{P}_{jk}{+a}_{5}{K}_{k}+{\varepsilon }_{ijk}^{a}.$$

#### Model 4: with past care markers and with provider effects

Finally, we consider both past care markers and provider effects as observed variables. Equation 1 then reflects the true cost and can be estimated as a function of observed variables:

11 $${C}_{ijk}={c}_{0}+{c}_{1}{x}_{1 ijk}+{c}_{2}{x}_{3ijk}+{c}_{3}{x}_{4jk}+{c}_{4}{z}_{jk}+{c}_{5}{P}_{jk}{+c}_{6}{K}_{k}+{\varepsilon }_{ijk}^{c}.$$

The probability limits of each coefficient $\widehat{d}$, $\widehat{g}$, $\widehat{a}$, $\widehat{c}$ are reported in Table 1 (columns 1, 2, 3 and 4, respectively). *b*_s_ are the probability limits of the coefficients from the regression of the unobserved variables *x*_*2*_ and *s* over each observed variable (in turn *x*_*1*_, *x*_*4*_, *z*, *K*), conditional to the remaining observed ones.

### Omitted variable bias and its relevance for need predictions

Comparing the probability limits of the coefficients in each of the four models (Table 1) allows us to illustrate potential omitted variables bias and its direction.

**Table 1:** Probability limits of the coefficients in Models 1 to 4

	Model 1 Without past care markers and without provider effects		Model 2 With past care markers and without provider effects		Model 3 Without past care markers and with provider effects		Model 4 With past care markers and with provider effects	
	Plim	Coefficient	Plim	Coefficient	Plim	Coefficient	Plim	Coefficient
Constant	d_0_	${\beta }_{0}+{\beta }_{3}{\varphi }_{0}+({\beta }_{3}{\varphi }_{5}+{\beta }_{6}){\xi }_{0}$	g_0_	${\beta }_{0}+{\beta }_{6}{\xi }_{0}$	a_0_	${\beta }_{0}+{\beta }_{3}{\varphi }_{0}$	c_0_	${\beta }_{0}$
x_1ijk_	d_1_	${\beta }_{1}+{\beta }_{3}{\varphi }_{1}+\left({\beta }_{2}+{\beta }_{3}{\varphi }_{2}\right){b}_{x2x1.x4zK}+({{\beta }_{3}\varphi }_{5}+{\beta }_{6}){\xi }_{3}{b}_{sx1.x4zK}$	g_1_	${\beta }_{1}+{\beta }_{2}{b}_{x2x1.x3x4zK}+{\beta }_{6}{\xi }_{3}{b}_{sx1.x3x4zK}$	a_1_	${\beta }_{1}+{\beta }_{3}{\varphi }_{1}+$ $({\beta }_{2}+{\beta }_{3}{\varphi }_{2}){b}_{x2x1.x4zPK}$	c_1_	${\beta }_{1}+{\beta }_{2}{b}_{x2x1.x3x4zPK}$
x_3ijk_			g_2_	${\beta }_{3}+{\beta }_{2}{b}_{x2x3.x1x4zK}+{\beta }_{6}{\xi }_{3}{b}_{sx3.x1x4zK}$			c_2_	${\beta }_{3}+{\beta }_{2}{b}_{x2x3.x1x4zPK}$
x_4ijk_	d_2_	${\beta }_{4}+{\beta }_{3}{\varphi }_{3}+({\beta }_{3}{\varphi }_{5}+{\beta }_{6}){\xi }_{1}+\left({\beta }_{2}+{\beta }_{3}{\varphi }_{2}\right){b}_{x2x4.x1zK}+({{\beta }_{3}\varphi }_{5}+{\beta }_{6}){\xi }_{3}{b}_{sx4.x1zK}$	g_3_	${\beta }_{4}+{\beta }_{6}{\xi }_{1}+{\beta }_{2}{b}_{x2x4.x1x3zK}+{\beta }_{6}{\xi }_{3}{b}_{sx4.x1x3zK}$	a_2_	${\beta }_{4}+{\beta }_{3}{\varphi }_{3}+$ $({\beta }_{2}+{\beta }_{3}{\varphi }_{2}){b}_{x2x4.x1zPK}$	c_3_	${\beta }_{4}+{\beta }_{2}{b}_{x2x4.x1x3zPK}$
z_jk_	d_3_	${\beta }_{5}+{\beta }_{3}{\varphi }_{4}+({\beta }_{3}{\varphi }_{5}+{\beta }_{6}){\xi }_{2}+({\beta }_{2}+{\beta }_{3}{\varphi }_{2}){b}_{x2z.x1x4K}+({{\beta }_{3}\varphi }_{5}+{\beta }_{6}){\xi }_{3}{b}_{sz.x1x4K}$	g_4_	${\beta }_{5}+{\beta }_{6}{\xi }_{2}+{\beta }_{2}{b}_{x2z.x1x3x4K}+{\beta }_{6}{\xi }_{3}{b}_{sz.x1x3x4K}$	a_3_	${\beta }_{5}+{\beta }_{3}{\varphi }_{4}+$ $({\beta }_{2}+{\beta }_{3}{\varphi }_{2}){b}_{x2z.x1x4PK}$	c_4_	${\beta }_{5}+{\beta }_{2}{b}_{x2z.x1x3x4PK}$
P_jk_					a_4_	${\beta }_{6}+{\beta }_{3}{\varphi }_{5}+({\beta }_{2}+{\beta }_{3}{\varphi }_{2}){b}_{x2P.x1x4zK}$	c_5_	${\beta }_{6}+{\beta }_{2}{b}_{x2P.x1x3x4zK}$
K_k_	d_4_	${\beta }_{7}+{\beta }_{3}{\varphi }_{6}+({\beta }_{3}{\varphi }_{5}+{\beta }_{6}){\xi }_{4}+({\beta }_{2}+{\beta }_{3}{\varphi }_{2}){b}_{x2K.x1x4z}+({{\beta }_{3}\varphi }_{5}+{\beta }_{6}){\xi }_{3}{b}_{sK.x1x4z}$	g_5_	${\beta }_{6}{\xi }_{4}+{\beta }_{7}+{\beta }_{2}{b}_{x2K.x1x3x4z}+{\beta }_{6}{\xi }_{3}{b}_{sK.x1x3x4z}$	a_5_	${\beta }_{7}+{\beta }_{3}{\varphi }_{6}+$ $({\beta }_{2}+{\beta }_{3}{\varphi }_{2}){b}_{x2K.x1x4zP}$	c_6_	${\beta }_{7}+{\beta }_{2}{b}_{x2K.x1x3x4zP}$
Error	${\varepsilon }_{ijk}^{d}$	$\left({\beta }_{3}{\varphi }_{5}+{\beta }_{6}\right){\varepsilon }_{jk}^{P}$ $+{{\beta }_{3}\varepsilon }_{ijk}^{x3} +{\varepsilon }_{ijk}$	${\varepsilon }_{ijk}^{g}$	${{\beta }_{6}\varepsilon }_{jk}^{P}+{\varepsilon }_{ijk}$	${\varepsilon }_{ijk}^{a}$	${\beta }_{3}{\varepsilon }_{ijk}^{x3}+{\varepsilon }_{ijk}$	${\varepsilon }_{ijk}^{c}$	${\varepsilon }_{ijk}$

The coefficient on individual need variables *x*_*1ijk*_ in Model 1 is composed by the direct effect of *x*_*1ijk*_ on cost and three types of indirect effects which depend on the correlation between *x*_*1ijk*_ and the unobserved *x*_*2ijk,*_* x*_*3ijk,*_ and *s*_*jk*_ and which may generate bias:

- the indirect effect through the unobserved past care markers *x*_*3ijk*_;
- the indirect effect of the unobserved individual need *x*_*2ijk*_ through *x*_*1ijk*_ and *x*_*3ijk*_, if *x*_*2ijk*_ was correlated with *x*_*1ijk*_ so that *b*_*x2x1.x4zK*_ was not 0;
- the indirect effect of the unobserved supply *s*_*jk*_ through *x*_*3ijk*_ and *P*_*jk*_, if *s*_*jk*_ was correlated with *x*_*1ijk*_ so that *b*_*sx1.x4zK*_ was not 0.

For resource allocation purposes, the bias would generate concerns only if *b*_*sx1.x4zK*_ was not equal 0, so that the coefficient would pick up unobserved supply which would then inappropriately be reflected in need weights. The bias from *b*_*sx1.x4zK*_ would not arise in Model 3 and Model 4, which include *P*_*jk*_ to further control for supply.

Similarly the coefficient estimates on GP practice need variables *x*_*4ijk*_, could be biased in Model 1 and Model 2 if *x*_*4ijk*_ was correlated with unobserved supply *s*_*jk*_, so that *b*_*sx4.x1zK*_ in Model 1 and *b*_*sx4.x1x3zK*_ in Model 2 were not 0. The bias in the coefficient estimated in Model 1 would also be a concern if past care markers were correlated with unobserved supply, so that ${\varphi }_{5}$ was not 0 and the coefficient would reflect the effect of unobserved supply on past care markers.

The estimated coefficients on GP practice observed supply variables, *z*_*jk*_, could be biased and of potential concern, in Models 1 and 3, where the past care markers *x*_*3ijk*_ were unobserved and correlated with *z*_*jk*_ (${\beta }_{3}{\varphi }_{4}$ not equal to 0). In Models 1 to 4 if supply was correlated with other unobserved individual need (*b*_*x2z*_ not equal to 0) coefficients would also be biased. This bias would raise concerns if unobserved individual need differed systematically across areas because when predicting need systematic variations in unobserved need would be sterilised together with the effect of GP practice supply. Models 2 and 4 would have smallest bias, if *b*_*x2z*_ was not 0, while Model 4 would have the smallest bias if also ${\beta }_{3}{\varphi }_{4}$ was not 0, as the latter effect would be picked up directly by past care markers.

The coefficients on the CCG fixed effects are larger in Model 1, 2 and 3 as they pick up the average CCG effect of unobserved supply and need variables. Because CCG fixed effects are considered supply factors and their effect sterilised in need predictions, the bias in their coefficients is of potential concern only if it reflects unobserved need. This would be the case in Model 1 and in Model 3, if past care markers were associated with unobserved GP practice and CCG supply, and if ${\varphi }_{5}$ and ${\varphi }_{6}$ were not 0. If unobserved need differed systematically across CCGs, *b*_*x2K*_ would not be 0 in Models 1 to 4 and the estimates of CCG fixed effects would be biased in every model, but with the smallest bias in Model 4.

The inclusion of the past care markers *x*_*3ijk*_ in Models 2 and 4 improves the precision of the coefficients on the remaining observed need and supply variables. The estimated coefficients on *x*_*3ijk*_ includes the direct effect of past care markers and other unobserved individual need variables. Crucially, if past care markers were correlated with unobserved supply, *b*_*sx3*_ would not be 0, and the coefficient estimated on *x*_*3ijk*_ in Model 2 would reflect supply differences, such as the propensity of different providers to diagnose, treat and record. This would not be the case in Model 4, where the provider effects would account for those supply effects.

The inclusion of provider effects *P*_*jk*_ in Model 2 and 4 improves the precision of the coefficients on the remaining need and supply variables. The coefficient on *P*_*jk*_ is larger in Model 3 when past care markers are not included,

If provider effects *P*_*jk*_ are correlated with unobserved individual need *x*_*3ijk*_ differing systematically at the area level, the coefficient on *P*_*jk*_ may reflect area differences in unobserved individual need, similarly to other observed supply variables.

The size of the unexplained variation is expected to reduce from Model 1 to Model 4 as more variables are included.

## Data

We used the dataset produced for the refreshment of the resource allocation formula for adult secondary mental health care in England [23]. This includes routinely available person level data on use and cost of mental health care services in 2015 linked with need and supply individual, area and GP practice variables in the two previous years (financial years, 1 April to 31 March) for all 43,750,558 patients over 20 years of age registered with a GP practice in England. Further details on data sources, extraction and linkage are provided elsewhere [23].

Person level data included mental health care contacts in outpatient settings and in the community, costed by the pay band of the professional providing care, and inpatient bed days, costed by level of intensity. Psychological therapies were included. Specialised services that are nationally commissioned (such as bed days in low, medium and high security wards) were excluded. The individual total annual cost was truncated at £100,000 affecting less than 0.01% of all individuals.

We included demographics and household composition (defined according to age and gender of individuals residing at the same address), ethnicity and physical health diagnoses associated with severe mental illness [38], which we use as observed characteristics due to reliable data quality.

Past care markers were derived from the Mental Health Services Dataset (MHSDS) and include diagnoses and intensity of previous care contacts. We defined a set of binary indicators for whether the person had been in contact with secondary mental health services and diagnosed with any of the 44 ICD-10 chapter F mental health diagnoses in 2013/14 or 2014/15. We generated a set of binary risk indicators, adapted from previous mental health formulae [31], including: the frequency of contacts with six different types of health care professionals defined by pay bands; an inpatient stay of two or more nights in a mental health Trust; a stay in a secure mental health ward; and a detention under the Mental Health Act.

We included GP practice or small geographical area (Lower level Super Output Area (LSOA)) level need variables were measured in 2013/14 or 2014/15: the proportion of the LSOA population receiving out of work benefit; a binary indicator for the GP practice serving a high proportion of students; and the GP practice prevalence of severe mental illness. Attributed supply variables included the driving time distance between the LSOA and closest Mental Health Trust headquarters.

We generated two sets of binary variables for the 211 CCGs and for the 42 higher-level STPs of which the GP practice were part. We also generated a set of provider effects, calculated as the proportion of patients registered with a given GP practice and who were in contact at least once with each of the 66 Trusts providing mental health care and reporting to the MHSDS over the financial years 2013 or 2014. As an alternative, we also calculated the two components of provider effects separately as: contact with any provider (proportion of patients registered with a given GP practice who were in contact at least once with at least one Trust) and contact with a specific provider (proportion in contact with each provider out of those patients who had at least one contact with one provider).

## Empirical analysis

### Model estimation

We estimated individual mental health care costs as a function of need and supply variables using a cross-section linear regression model (Ordinary Least Squares) with robust standard errors and CCG fixed effects. We included binary indicators for the interactions between gender and 14 five-year age bands (up to 85 +), 16 ethnic groups, 11 household types and 7 physical health diagnoses, as person level measures of need. We also included GP practice (or area) need and supply variables. The CCG dummies control for residual differences in supply.

We estimated the four models presented in Sect. 3.2. We first estimated a base model where we included all individual and area need and supply variables and CCG dummies, but no past care markers nor provider effects (Eq. 6, Model 1). We then additionally included past care markers (Eq. 8, Model 2) or provider effects (Eq. 10, Model 3), or both (Eq. 11, Model 4). For clarity of exposition we have not included time subscripts, but in the empirical models all need and supply variables were measured historically to avoid reverse causality.

For each model, we estimated the mental health care cost for all individuals registered with a random 50% of 7746 GP practices and we used the remaining observations as a validation sample for the predictions. We compared the models’ predictive performance based on the coefficient of variation (R-squared) at the individual and at the GP practice level on both the estimation and validation samples. Model selection was based on: predictive power (at the GP practice, rather than person level and on both estimation and valuation samples); variation of coefficients when including or excluding variables; and redistributive effects of alternative models, in line with international practice [9]. The objective of the formula is to allocate resources at the commissioner level, but the predictive power of the model cannot be assessed at this level given the inclusion of commissioners fixed effects. The performance is therefore assessed at the GP practice level [16]. Assessment of the performance of the model in explaining individual-level variation serves to understand the effects of including different variables and to check coefficient stability.

### Coefficient stability

Coefficient stability when including additional covariates is generally interpreted as a sign of limited omitted variable bias. However, small coefficient movement could be due to the low explanatory power of these additional covariates [25]. Oster [25] suggests that coefficient movements should be interpreted alongside R-squared movements and proposes a consistent estimator of the bias which accounts for both. She derives a bias correction using the coefficient and R-squared from a baseline regression without observable controls ($$\dot{\beta }$$ and $$\dot{R}$$); the coefficient and R-squared from a regression with observable controls ($$\overset{\lower0.5em\hbox{$\smash{\scriptscriptstyle\smile}$}}{\beta }$$ and $$\overset{\lower0.5em\hbox{$\smash{\scriptscriptstyle\smile}$}}{R}$$); and the R-squared from an hypothetical regression which maximises R-squared (*R*_*max*_).

Although the maximum R-squared would be 1, Oster [25] recognises that in practice no regression can fully explain the observed variation and suggests using a value proportional to $$\overset{\lower0.5em\hbox{$\smash{\scriptscriptstyle\smile}$}}{R}$$ as an upper bound for *R*_*max*_. The estimator is derived under the assumptions of equal selection on observables and unobservables, and of equal contributions to the variable of interest and to the outcome of each observable control. Oster [25] suggests that the unbiased coefficient lies within the bounds [$${\upbeta }^{*},$$ $$\overset{\lower0.5em\hbox{$\smash{\scriptscriptstyle\smile}$}}{\beta }$$]. The smaller these bounds, the smaller the coefficient bias.

Following Oster [25], we derived the bias corrected coefficients for need and supply variables, including past care markers, as:

12 $$ {\upbeta }^{*} = \overset{\lower0.5em\hbox{$\smash{\scriptscriptstyle\smile}$}}{\beta } - \left[ {\dot{\beta } - \overset{\lower0.5em\hbox{$\smash{\scriptscriptstyle\smile}$}}{\beta } } \right]\frac{{R_{{{\text{max}}}} - \overset{\lower0.5em\hbox{$\smash{\scriptscriptstyle\smile}$}}{R} }}{{\overset{\lower0.5em\hbox{$\smash{\scriptscriptstyle\smile}$}}{R} - \dot{R}}}, $$

where $$\overset{\lower0.5em\hbox{$\smash{\scriptscriptstyle\smile}$}}{\beta }$$ and $$\overset{\lower0.5em\hbox{$\smash{\scriptscriptstyle\smile}$}}{R}$$ were derived from Model 4, which included the most complete set of observables with provider effects, $$\dot{\beta }$$ and $$\dot{R}$$ were derived from Model 2, which included a limited set of observables and *R*_max_ is the maximum R-squared attainable. Unlike Oster [25] we didn’t use *R*_max_ = 1.3 $$\overset{\lower0.5em\hbox{$\smash{\scriptscriptstyle\smile}$}}{R}$$, but we set *R*_max_ = 0.2753, the R-squared of the individual level regression including GP practice fixed effects (R-squared 0.2755, Adjusted R-Squared 0.2753). GP practice fixed effects would maximise R-squared at the GP practice, not person, level, which is the main metric used to compare models predictive power in the Person Based Resource Allocation methodology [16]. Given the still limited set of individual need variables, GP practice fixed effects are not included in any of the models as their coefficients cannot be interpreted exclusively as need or supply, and they would prevent the inclusion of other time invariant GP practice need and supply variables.

### Sensitivity to variation in provider use within purchaser

Given the number of Trusts (66 providers) compared to CCGs (211 local purchasers), one may expect little variation in provider effects within CCGs, but higher variations of provider effects within STPs (42 groups of CCGs). We tested whether results are sensitive to allowing for higher variation of provider effects within purchaser by including STP instead of CCG fixed effects and we re-estimated Models 1 to 4 as Models 5 to 8. Table 5 in the Appendix reports the number of CCGs within different STPs.

### Sensitivity to variations in the definition of provider effects

The two components of the provider effects (contact with any provider and contact with a specific provider) may have different effects on mental health care cost. We tested if controlling for them separately increases precision in the estimate of the coefficients of interest. We re-estimated Models 3, 4, 7 and 8 as Models 9, 10, 11 and 12 by including contact with any provider and contact with a specific provider, instead of the provider effects originally defined as proportions of GP practice patients in contact with each provider.

### Sensitivity to provider data reporting

As a further sensitivity analysis, we estimated Models 1, 2, 3 and 4 excluding CCGs where the majority of patients (> 95%) used Trusts with the lowest reporting quality, assessed based on submissions with missing data for 2013, 2014 and 8 months of 2015 [34, 35].

## Results

### Descriptive statistics

Out of the 43,750,558 adults aged 20 years or older, 4.01% had some contact with secondary mental health and/or IAPT services in 2015/2016. The average cost per person was £80.60, whilst the cost per service user was £ 2008.46 on average and ranged from £ 94 to £ 1,040,963 (before truncation). The descriptive statistics are reported in Table 6 in the Appendix.

### Importance of past care markers

Table 2 presents the coefficients estimated in Models 1, 2, 3 and 4 and the Oster’s bias corrected coefficient on some examples of individual and GP practice variables included in the models. The complete sets of coefficients for all estimated models are reported in Table 6 in the Appendix.

**Table 2:** Coefficients on some need and supply variables

	Without past care markers and without provider effects (Model 1)	With past care markers and without provider effects (Model 2)	Without past care markers and with provider effects (Model 3)	With past care markers and with provider effects (Model 4)	Oster bias corrected coefficient (Model 4 vs Model 2)
Person characteristics (examples)					
25–29 years Female	− 0.634 (− 0.328)	0.290 (0.182)	− 0.455 (− 0.235)	0.315 (0.197)	0.34
35–39 years Male	13.812*** (6.123)	− 5.170** (− 2.745)	14.089*** (6.246)	− 5.172** (− 2.746)	− 5.174
Irish	33.920*** (5.076)	5.690 (1.030)	33.914*** (5.076)	5.628 (1.019)	5.566
Any other White	− 24.768*** (− 13.205)	− 13.151*** (− 8.236)	− 24.562*** (− 13.078)	− 13.175*** (− 8.238)	− 13.199
White and Black Caribbean	139.081*** (8.456)	37.990** (2.722)	140.276*** (8.531)	38.499** (2.760)	39.008
At least one admissions with viral hepatitis (B15-B19)	286.099*** (6.960)	41.532 (1.158)	284.688*** (6.931)	41.392 (1.155)	41.252
At least one admissions with Cerebrovascular diseases (I60-I69)	52.323*** (4.167)	21.027 (1.917)	52.102*** (4.150)	20.922 (1.907)	20.817
Living in care home	437.190*** (32.624)	18.354 (1.580)	436.937*** (32.597)	19.632 (1.688)	20.91
Living in two adults of same gender household	29.742*** (14.773)	8.027*** (4.711)	29.895*** (14.851)	8.013*** (4.703)	7.999
Past care markers (examples)					
Had an admission in a medium secure ward		6265.422*** (11.912)		6258.963*** (11.909)	6252.504
Has been detained under the mental health act		119.907 (0.813)		119.916 (0.813)	119.925
At least 2 contacts with 2 different types professionals		4911.304*** (131.829)		4911.591*** (131.869)	4911.878
At least 3 contacts with 3different types professionals		11,380.122*** (101.142)		11,380.710*** (101.155)	11,381.298
F00—(Dementia in Alzheimer's disease)		280.413*** (7.553)		281.595*** (7.581)	282.777
F01—(Vascular dementia)		144.636 (1.863)		146.709 (1.890)	148.782
Area level need					
Rate receiving out of work benefit	266.678*** (34.636)	78.655*** (12.189)	269.048*** (34.964)	75.579*** (11.702)	72.503
Student GP practice	− 25.557*** (− 8.691)	− 17.038*** (− 6.956)	− 28.164*** (− 8.990)	− 18.725*** (− 7.159)	− 20.412
MH prevalence rate	46.551*** (21.387)	15.072*** (8.484)	22.323*** (11.290)	6.468*** (3.912)	− 2.136
Area level supply					
Drive time to nearest mental health provider headquarter	− 0.362*** (− 10.078)	− 0.194*** (− 6.241)	− 0.331*** (− 8.774)	− 0.224*** (− 6.831)	− 0.254
Local Health Organisation (CCGs) fixed effects	Yes	Yes	Yes	Yes	Yes
Provider effects	No	No	Yes	Yes	Yes
R2 individual level	0.0073	0.2746	0.0075	0.2747	
R2 GP practice level—Estimation	0.723	0.8101	0.8078	0.8648	
R2 GP practice level—Verification	0.7203	0.8155	0.7929	0.8481	

Observations 21,319,709; robust t statistics in parentheses; *p < 0.05; **p < 0.01; ***p < 0.001; *R*_max_ used in the calculation of Oster bias corrected coefficient is 0.2753. The table reports coefficients for examples of variables from the sets of person characteristics (age, gender, ethnicity and household type) and past care markers (ICD-10 chapter F mental health diagnoses and risk indicators). The online Appendix reports coefficients for all variables

As expected, compared with Model 1, the coefficients on individual characteristics in Model 2 and Model 4 are affected by the introduction of the past care markers, which now account for unobserved individual need. Coefficients are similar in Model 1 and in Model 3 after the inclusion of provider effects, except for some age groups, suggesting that unobserved GP practice need and supply are controlled for.

Changes in the coefficients on area and GP practice need variables indicate that in Model 1 they picked-up need which is picked-up by past care markers in Model 2. The inclusion of provider effects in Model 3 reduces the coefficients on these variables compared with Model 1, where the coefficients may also pick-up the effect of some unobserved GP practice need and supply, if these are not appropriately controlled for by the remaining variables included. The inclusion of provider effects in Model 4 does not change the coefficients compared with Model 2, suggesting that when individual need is controlled for, provider effects are not accounting for GP practice need, but rather for supply.

The coefficients on area supply variables are affected substantially by the inclusion of past care markers in Model 2, and marginally by the inclusion of provider effects in Model 3. If past care markers are not included, coefficients on area supply variables may reflect unobserved differences in need, then sterilised when predicting need.

The inclusion of past care markers in Model 2 improves the R-squared from 0.0073 to 0.2746 at the individual level and from 0.7203 to 0.8155 at the GP practice level.

### Importance of provider effects

Provider effects in Model 3 and Model 4 pick-up variation in supply across GP practices within CCGs and reduce the size and precision of the coefficients on CCG dummies. The reduction is larger from Model 1 to Model 3, where past care markers are not included, than from Model 2 to Model 4, indicating that provider effects pick-up variation in unobserved need otherwise picked-up by CCG dummies. The reduction in CCG dummies coefficients after including past care markers is smaller when provider effects are also included (from Model 3 to Model 4 rather than from Model 1 to Model 2). Moreover, the inclusion of past care markers tends to reduce the size of the coefficients on the provider effects from Model 3 to Model 4. These are indications that provider effects account for variation in unobserved need at the area level when it is not accounted for by individual controls. The inclusion of provider effects increases R-squared at the GP practice level only, from 0.7203 in Model 1 to 0.7929 in Model 3 without past care markers, and from 0.8155 in Model 2 to 0.8481 in Model 4 with past care markers.

### Robustness checks

The coefficients on past care markers in Model 2 are not affected by the inclusion of provider effects in Model 4. If past care markers were correlated to unobserved differences in supply and recording across providers, these were already controlled for by the CCG fixed effects. The Oster bias corrected coefficients are very close to the coefficients estimated in Model 4, making the bounds for the true coefficient very narrow. Coefficients on the past care markers are stable even when their additional explanatory power is accounted for through R-squared.

When replicating the analysis using higher level purchaser fixed effects (STPs rather than CCGs) allowing for higher variation in provider use, results remain unchanged for individual need variables, but coefficients on area and GP practice variables are smaller. Table 3 presents examples of coefficients estimated in Models 5 to 8 using STP dummies. The full set is presented in the Online Appendix.

**Table 3:** Coefficients from models with STP dummies

	Without past care markers and without provider effects (Model 5)	With past care markers and without provider effects (Model 6)	Without past care markers and with provider effects (Model 7)	With past care markers and with provider effects (Model 8)	Oster bias corrected coefficient (Model 8 vs Model 6)
Person characteristics (examples)					
25–29 years Female	− 0.744 (− 0.385)	0.064 (0.040)	− 0.428 (− 0.222)	0.445 (0.279)	0.826
35–39 years Male	13.914*** (6.168)	− 5.208** (− 2.765)	14.223*** (6.304)	− 4.998** (− 2.653)	− 4.788
Irish	33.655*** (5.035)	3.790 (0.686)	33.770*** (5.053)	5.451 (0.987)	7.112
Any other White	− 26.231*** (− 14.100)	− 15.209*** (− 9.606)	− 24.047*** (− 12.879)	− 12.562*** (− 7.903)	− 9.915
White and Black Caribbean	139.747*** (8.505)	36.102** (2.589)	140.200*** (8.529)	39.269** (2.816)	42.436
At least one admissions with viral hepatitis (B15-B19)	286.428*** (6.968)	42.272 (1.179)	285.610*** (6.955)	41.762 (1.166)	41.252
At least one admissions with Cerebrovascular diseases (I60-I69)	52.372*** (4.172)	20.515 (1.870)	51.891*** (4.134)	20.607 (1.879)	20.699
Living in care home	437.514*** (32.645)	18.390 (1.583)	436.838*** (32.593)	19.809 (1.704)	21.228
Living in two adults of same gender household	29.739*** (14.779)	7.976*** (4.683)	29.957*** (14.889)	8.191*** (4.810)	8.406
Past care markers (examples)					
Had an admission in a medium secure ward		6266.636*** (11.915)		6259.143*** (11.911)	6251.65
Has been detained under the mental health act		119.749 (0.812)		119.617 (0.811)	119.485
At least 2 contacts with 2 different types professionals		4908.773*** (131.771)		4911.637*** (131.869)	4914.501
At least 3 contacts with 3different types professionals		11,375.212*** (101.094)		11,380.774*** (101.155)	
F00—(Dementia in Alzheimer's disease)		279.040*** (7.517)		281.560*** (7.581)	11,386.336
F01—(Vascular dementia)		144.140 (1.857)		146.840 (1.892)	149.54
Area level need					
Rate receiving out of work benefit	245.298*** (33.313)	70.859*** (11.496)	248.947*** (33.285)	67.478*** (10.753)	64.097
Student GP practice	− 18.660*** (− 7.182)	− 11.048*** (− 5.121)	− 27.993*** (− 10.387)	− 15.590*** (− 6.945)	− 20.132
MH prevalence rate	43.889*** (22.792)	12.416*** (7.901)	21.398*** (11.487)	7.204*** (4.637)	1.992
Area level supply					
Drive time to nearest mental health provider headquarter	− 0.135*** (− 5.339)	− 0.152*** (− 6.990)	− 0.213*** (− 7.217)	− 0.145*** (− 5.604)	− 0.138
STP fixed effects	Yes	Yes	Yes	Yes	Yes
Provider effects	Yes	Yes	Yes	Yes	Yes
R2 Individual level	0.0073	0.2746	0.0076	0.2748	
R2 GP practice level—Estimation	0.655	0.7536	0.7853	0.8513	
R2 GP practice level—Verification	0.6891	0.7957	0.7997	0.8592	

Observations 21,319,709; robust t statistics in parentheses; *p < 0.05; **p < 0.01; ***p < 0.001; *R*_max_ used in the calculation of Oster bias corrected coefficient is 0.2753.The table reports coefficients for examples of variables from the sets of person characteristics (age, gender, ethnicity and household type) and past care markers (ICD-10 chapter F mental health diagnoses and risk indicators).Table 6 in Appendix reports coefficients for all variables

Table 4 presents the coefficients for models 3, 4, 7 and 8 re-estimated as Models 9, 10, 11, 12 including contact with any provider and contact with a specific provider as two separate components of provider effects. Results are unchanged for individual variables, while the size of coefficients on area and GP practice need and supply variables is affected, suggesting that coefficients on need variables may have been biased by supply. R-squared is unaffected.

**Table 4:** Coefficients from models with alternative provider effects

	Without past care markers and with provider effects (Model 9)	With past care markers and with provider effects (Model 10)	Oster bias corrected coefficient (Model 10 vs Model 2)	Without past care markers and with provider effects (Model 11)	With past care markers and with provider effects (Model 12)	Oster bias corrected coefficient (Model 12 vs Model 6)
Person characteristics (examples)						
25–29 years Female	− 0.413 (− 0.214)	0.458 (0.287)	0.2228	− 0.526 (− 0.272)	0.483 (0.303)	0.902
35–39 years Male	14.137*** (6.268)	− 5.030** (− 2.671)	− 5.226	14.062*** (6.233)	− 5.012** (− 2.661)	− 4.816
Irish	34.509*** (5.165)	5.767 (1.044)	5.6592	33.926*** (5.077)	5.464 (0.989)	7.138
Any other White	− 24.360*** (− 12.963)	− 12.926*** (− 8.080)	− 13.241	− 23.965*** (− 12.807)	− 12.513*** (− 7.857)	
White and Black Caribbean	140.319*** (8.532)	38.424** (2.754)	37.8164	140.501*** (8.547)	39.055** (2.800)	− 9.817
At least one admissions with viral hepatitis (B15-B19)	285.746*** (6.958)	41.913 (1.170)	41.3796	285.899*** (6.961)	42.127 (1.176)	41.982
At least one admissions with Cerebrovascular diseases (I60-I69)	52.149*** (4.153)	20.996 (1.914)	21.0394	52.285*** (4.165)	20.762 (1.893)	21.009
Living in care home	436.140*** (32.549)	18.460 (1.589)	18.3116	436.260*** (32.560)	18.605 (1.602)	
Living in two adults of same gender household	29.850*** (14.827)	8.049*** (4.724)	8.0182	29.974*** (14.897)	8.224*** (4.829)	18.82
Past care markers (examples)						
Had an admission in a medium secure ward		6260.082*** (11.909)	6267.558		6260.917*** (11.911)	6255.198
Has been detained under the mental health act		120.249 (0.815)	119.7702		119.837 (0.812)	119.925
At least 2 contacts with 2 different types professionals		4911.678*** (131.858)	4911.1544		4911.739*** (131.860)	
At least 3 contacts with 3different types professionals			11,379.96		11,380.526*** (101.151)	4914.705
F00—(Dementia in Alzheimer's disease)		11,380.527*** (101.151) (7.543)	280.563		279.958*** (7.541)	280.876
F01—(Vascular dementia)		144.183 (1.858)	144.8172		144.157 (1.857)	144.174
Area level need						
Rate receiving out of work benefit	259.054*** (32.299)	74.718*** (11.083)	80.2298	243.493*** (31.425)	69.279*** (10.643)	67.699
Student GP practice	− 28.126*** (− 9.032)	− 19.349*** (− 7.440)	− 16.1136	− 28.131*** (− 10.124)	− 18.053*** (− 7.795)	− 25.058
MH prevalence rate	29.705*** (10.631)	7.317** (3.213)	18.174	28.333*** (11.399)	8.532*** (4.213)	4.648
Area level supply						
Drive time to nearest mental health provider headquarter	− 0.333*** (− 8.701)	− 0.220*** (− 6.636)	− 0.1836	− 0.190*** (− 6.478)	− 0.130*** (− 5.097)	− 0.108
CCG fixed effects	Yes	Yes	Yes	No	No	No
STP fixed effects	No	No	No	Yes	Yes	Yes
GP Contact with any provider	743.698*** (6.199)	222.493* (2.190)	Yes	788.091*** (7.407)	163.056 (1.809)	Yes
GP Contact with specific providers	Yes	Yes	Yes	Yes	Yes	Yes
R2 Individual level	0.0075	0.2747		0.2671	0.2748	
R2 GP practice level—Estimation	0.7858	0.8532		0.8363	0.8383	
R2 GP practice level—Verification	0.764	0.8379		0.8505	0.8549	

Observations 21,319,709; robust t statistics in parentheses; *p < 0.05; **p < 0.01; ***p < 0.001; *R*_max_ used in the calculation of Oster bias corrected coefficient is 0.2753.The table reports coefficients for examples of variables from the sets of person characteristics (age, gender, ethnicity and household type) and past care markers (ICD-10 chapter F mental health diagnoses and risk indicators). The Online Appendix reports coefficients for all variables

When estimating Model 2 and Model 4 excluding CCGs served by low reporting quality Trusts, the coefficients remained unchanged (Model 12 and Model 16 are reported in the Online Appendix). This suggests that CCG dummies and provider controls account for provider variation and coefficients are not biased by reporting differences.

## Discussion

We have illustrated how the inclusion of care markers from past care contacts in risk-adjustment models can increase predictive power and disentangle the contributions of need and supply factors to variations in costs. The inclusion of provider effects can account for the potential correlation between past care markers and systematic differences in diagnostic, treatment and recording quality. We illustrated the issue using the example of the risk-adjustment model developed for the latest English resource allocation formula for mental health. The inclusion of past mental care markers improves coefficient precision, irrespective of whether provider effects are included. The coefficients on the past care markers are stable across different model specifications, suggesting that any biases from variation in supply are sufficiently controlled for by fixed effects for purchaser organisations. The inclusion of provider effects improves the predictive power of the model and, alongside past care markers, contributes to disentangling need and supply drivers of variations in costs.

Our results build on the development of risk-adjustment formulae for resource allocation in England. We highlight how in person-based models, as in area level models [12], the inclusion of morbidity markers improves predictions and, alongside appropriate provider controls, ensures unbiased coefficients on the need variables. As discussed in Dixon et al. [37], when individual need variables are included in the model, past encounters and diagnoses at the area level tend to reflect supply rather than need. We show that past care use measured at the area or GP practice level serves as a control for unobserved supply variation across GP practices and providers within local health organisations. They effectively perform the same function as provider fixed effects. However, because not every patient uses the service and because patients can use multiple providers, their values vary between 0 and 1 across GP practices, and they reflect variations in total use as well as variation in the extent to which each provider is used.

With individual level data, we were able to refine the approach previously proposed at the area level [24] using individual past care markers and provider effects, alongside local health organisation fixed effects. We could also split provider effects into their GP practice and mental health trust components, which could both affect access to care. The inclusion of provider effects increased explained variation and improved the precision of the coefficients, but local health organisation fixed effects and GP practice supply variables are generally sufficient to avoid bias in the coefficients on individual-level need variables.

Results from the formal assessment of the bias in past care markers coefficients are relevant more broadly for risk-adjustment models based on administrative records from past care. It is well known that past care markers improves prediction, but there are still concerns about omitted variable bias [6]. Adapting the Oster [25] method to assess bias by evaluating coefficient stability according to variations in coefficient size and in R-squared, we showed that with sufficient control for supply factors the bias is minimal.

The use of diagnostic variables, and especially past care and expenditure may also provide perverse incentives and reproduce unfair utilisation patterns [6]. We showed that past care use at the area or GP practice level, if differentiated by provider can be helpful in controlling for variations in supply. In models that use standardisation techniques [39] to disentangle need and supply driven cost, the effect of area level past care use can be sterilised to control for any unobserved difference in supply. The approach is valid as long as there are enough variables capturing need at the individual level, such as in our example past mental health care markers. If those are not included, there is a risk of inappropriately sterilising legitimate differences in need.

We were able to carry out a highly comprehensive analysis including the whole adult population of England (over 40 million people), over 7500 primary care providers, 66 Mental Health Care providers and 211 purchasers. The large size of the dataset allowed the inclusion of a large number of covariates: 14 five-year age bands (up to 85 +), 16 ethnic groups, 11 household types and 7 physical health diagnoses, an additional 50 mental past care markers, along with GP practice and area level variables, 66 provider controls and 211 CCG fixed effects.

We were able to include a relatively large set of individual characteristics, compared to other studies based on administrative records. Indeed, individual ethnicity and household type were used for the first time in an analysis covering the whole population in England. We could then use area level past utilisation to control for variations in supply within local organisation, and rely on the individual, area and GP practice need variables to capture variations in need. The same methods and conclusions may not apply with smaller sample size which does not allow the inclusion of a similarly broad set of individual need variables and where administrative records may only be available for a limited set of providers or not cover the entire population.

A model including both individual past care markers and area controls for past provider contacts ideally produces the most equitable allocations. However, its implementation may be flawed unless appropriate techniques to impute unrecorded past care markers are developed and agreed. Because the formulae only aim at generating playing level field, it is important that it is complemented by other policies targeting different levels and aspects of service delivery which would also help to improve the reliability of past care markers. In settings like England, where most patients within the same local health organisation (CCG) use the same provider, the inclusion of local health organisation dummies could sufficiently control for unobserved supply bias in the coefficients on mental past care markers. However, the additional inclusion of provider controls contributes to minimising the bias in GP practice need variables. The choice between a model without past care markers and with or without providers care contacts depends on policy makers’ preferences over a trade-off. The model with provider care contacts, underestimates differences in need, but ensure that any unobserved difference in supply is controlled for. The model without provider care contacts increases the differentiation in estimated need, whilst accepting that some of it may reflect unobserved supply, but ensure that any unobserved difference in supply is controlled for.

### Supplementary Information

Below is the link to the electronic supplementary material.

Supplementary file1 (XLSX 214 KB)

## Appendix

See Tables 5 and 6.

**Table 5:** CCGs grouping into STP

STP	STP	Sustainability and Transformation Plan	CCG count
1	QHM	Cumbria and North East	12
4	QE1	Healthier Lancashire and South Cumbria	8
5	QWO	West Yorkshire and Harrogate (Health & Care Partnership)	11
6	QOQ	Humber, Coast and Vale	6
7	QOP	Greater Manchester Health and Social Care Partnership	12
8	QYG	Cheshire and Merseyside	12
9	QF7	South Yorkshire and Bassetlaw	5
10	QNC	Staffordshire and Stoke on Trent	6
11	QOC	Shropshire and Telford and Wrekin	2
12	QJ2	Joined Up Care Derbyshire	4
13	QJM	Lincolnshire	4
14	QT1	Nottingham and Nottinghamshire Health and Care	6
15	QK1	Leicester, Leicestershire and Rutland	3
16	QUA	The Black Country and West Birmingham	4
17	QHL	Birmingham and Solihull	3
18	QWU	Coventry and Warwickshire	3
19	QGH	Herefordshire and Worcestershire	4
20	QPM	Northamptonshire	2
21	QUE	Cambridgeshire and Peterborough	1
22	QMM	Norfolk and Waveney Health & Care Partnership	5
23	QJG	Suffolk and North East Essex	3
24	QHG	Bedfordshire, Luton and Milton Keynes	3
25	QM7	Hertfordshire and West Essex	3
26	QH8	Mid and South Essex	5
27	QRV	North West London Health & Care Partnership	8
28	QMJ	North London Partners in Health & Care	5
29	QMF	East London Health & Care Partnership	7
30	QKK	Our Healthier South East London	6
31	QWE	South West London Health & Care Partnership	6
32	QKS	Kent and Medway	8
33	QNX	Sussex and East Surrey	8
34	QNQ	Frimley Health & Care ICS	5
35	QXU	Surrey Heartlands Health & Care Partnership	3
36	QT6	Cornwall and the Isles of Scilly Health & Social Care Partnership	1
37	QJK	Devon	2
38	QSL	Somerset	1
39	QUY	Bristol, North Somerset and South Gloucestershire	3
40	QOX	Bath and North East Somerset, Swindon and Wiltshire	3
41	QVV	Dorset	1
42	QRL	Hampshire and the Isle of Wight	7
43	QR1	Gloucestershire	1
44	QU9	Buckinghamshire, Oxfordshire and Berkshire West	7

**Table 6:** Descriptive statistics

	Mean	Min	Max
Mental Health care user	0.04	0	1
Cost (£) per person	80.598	0	1,040,963
Cost (£) per user	2008.46	94	1,040,963
Male	0.493	0	1
Age			
20–24 years	0.082	0	1
25–29 years	0.091	0	1
30–34 years	0.093	0	1
35–39 years	0.086	0	1
40–44 years	0.089	0	1
45–49 years	0.094	0	1
50–54 years	0.09	0	1
55–59 years	0.077	0	1
60–64 years	0.068	0	1
65–69 years	0.07	0	1
70–74 years	0.053	0	1
75–79 years	0.042	0	1
80–84 years	0.032	0	1
85 years or older	0.035	0	1
Ethnicity			
White British	0.719	0	1
Irish	0.006	0	1
Any other White background	0.052	0	1
White and Black Caribbean	0.003	0	1
White and Black African	0.001	0	1
White and Asian	0.002	0	1
Any other mixed background	0.005	0	1
Indian	0.022	0	1
Pakistani	0.017	0	1
Bangladeshi	0.006	0	1
Any other Asian background	0.014	0	1
Caribbean	0.01	0	1
African	0.014	0	1
Any other Black background	0.007	0	1
Chinese	0.004	0	1
Any other ethnic group	0.022	0	1
Physical health diagnoses			
Viral hepatitis (ICD-10 codes B15-B19)	0.001	0	1
Symptoms and signs involving cognition, perception, emotional state and behaviour (ICD-10 codes R40-R46)	0.005	0	1
Poisoning by drugs, medicaments and biological substances (ICD-10 codes T36-T50)	0.002	0	1
Diabetes mellitus (ICD-10 codes E10-E14)	0.02	0	1
Endocrine nutritional and metabolic diseases (ICD-10 codes E15-E90)	0.029	0	1
Cerebrovascular diseases (ICD-10 codes I60-I69)	0.003	0	1
Chronic lower respiratory diseases (ICD-10 codes J40-J47)	0.03	0	1
Household type			
Care home	0.005	0	1
Missing	0.061	0	1
Multi-adult	0.254	0	1
Multi-adult and one or more children	0.123	0	1
Multi-child	0	0	1
Other communal	0.014	0	1
One adults and one or more children	0.024	0	1
Single person	0.132	0	1
Two adults and one or more children	0.13	0	1
Two adults of different gender (base category)	0.223	0	1
Two adults of the same gender	0.033	0	1
Attributed variables			
Need			
Proportion in LSOA receiving out of work benefits	0.088	0.001	0.49
Registered with Student GP practice	0.018	0	1
Prevalence (%) of Severe Mental Illness in GP practice	0.881	0.059	15.567
Supply			
Drive time to closest mental health trust (mins)	22.606	0.28	105.83
Mental past care markers			
Inpatient stay in a secure ward	0.000	0	1
Detained under MH act	0.000	0	1
At least 2 nights in a mental health hospital	0.003	0	1
At least 2 contacts with 2 professionals	0.004	0	1
At least 3 contacts with 3 professionals	0.001	0	1
At least 4 contacts with 4 professionals	0.000	0	1
At least 5 contacts with 5 professionals	0.000	0	1
At least 6 contacts with 6 professionals	0.000	0	1
F00—(Dementia in Alzheimer)	0.001	0	1
F01—(Vascular dementia)	0.000	0	1
F02—(Dementia in other diseases specified elsewhere)	0.000	0	1
F03—(Unspecified dementia)	0.000	0	1
F05—(Delirium not from alcohol)	0.000	0	1
F06—(Mental health disorder from brain damage)	0.001	0	1
F07—(Personality and behavioural dis from brain dis)	0.000	0	1
F10—(Mental and behavioural disorders due to use of alcohol)	0.001	0	1
F11—(Mental and behavioural disorders due to use of opioids)	0.000	0	1
F12—(Mental and behavioural disorders due to use of cannabis)	0.000	0	1
F13—(Mental and behavioural disorders due to use of sedatives or hypnotics)	0.000	0	1
F14—(Mental and behavioural disorders due to use of cocaine)	0.000	0	1
F15—(Mental and behavioural disorders due to use of other stimulants inc. caffeine)	0.000	0	1
F19—(Mental and behavioural disorders due to multiple drug/psychoactive sub use)	0.000	0	1
F20—(Schizophrenia)	0.001	0	1
F22—(Persistent delusional disorders)	0.000	0	1
F23—(Acute and transient psychotic disorders)	0.000	0	1
F25—(Schizoaffective disorders)	0.000	0	1
F29—(Unspecified nonorganic psychosis)	0.000	0	1
F30—(Manic episode)	0.000	0	1
F31—(Bipolar affective disorder)	0.001	0	1
F32—(Depressive episode)	0.001	0	1
F33—(Recurrent depressive disorder)	0.001	0	1
F34—(Persistent mood [affective] disorders)	0.000	0	1
F39—(Unspecified mood [affective] disorder)	0.000	0	1
F40—(Phobic anxiety disorders)	0.000	0	1
F41—(Other anxiety disorders)	0.001	0	1
F42—(Obsessive–compulsive disorder)	0.000	0	1
F43—(Reaction to severe stress and adjustment disorders)	0.001	0	1
F44—(Dissociative [conversion] disorders)	0.000	0	1
F45—(Somatoform disorders)	0.000	0	1
F50—(Eating disorders)	0.000	0	1
F60—(Specific personality disorders)	0.001	0	1
F61—(Mixed and other personality disorders)	0.000	0	1
F70—(Mild mental retardation)	0.000	0	1
F84—(Pervasive developmental disorders)	0.000	0	1
F90—(Hyperkinetic disorders)	0.000	0	1
F99—(Mental disorder otherwise specified	0.000	0	1
G30—(Alzheimer's disease)	0.000	0	1
G40—(Epilepsy)	0.000	0	1
R45—(Symptoms and signs involving emotional state)	0.000	0	1
Z81—(Family history of mental and behavioural disorders)	0.000	0	1
Z91—(Personal history of risk-factors)	0.001	0	1
